# The Convergence of Early-Life Stress and Autism Spectrum Disorder on the Epigenetics of Genes Key to the HPA Axis

**DOI:** 10.3390/biology15010066

**Published:** 2025-12-30

**Authors:** Edric Han, Katherine A. Canada, Meghan H. Puglia, Kevin A. Pelphrey, Tanya M. Evans

**Affiliations:** 1 Monroe Hall, College of Arts and Sciences, University of Virginia, Charlottesville, VA 22904, USA; eah6p@virginia.edu; 2 Gilmer Hall, Department of Neurology, University of Virginia, Charlottesville, VA 22904, USA; meghan.puglia@virginia.edu (M.H.P.); kap3n@uvahealth.org (K.A.P.); 3 Ridley Hall, School of Education and Human Development, University of Virginia, Charlottesville, VA 22904, USA; tme4j@virginia.edu

**Keywords:** autism spectrum disorder, gene–environment interactions, HPA axis, epigenetic regulation

## Abstract

Autism spectrum disorder (ASD) arises from complex genetic and environmental influences. Despite its prevalence and being the focus of study for several decades, its causes and their underlying mechanisms are still not fully understood. However, one consistent causal mechanism of interest is epigenetic modification. While some risk factors, such as maternal stress, nutrition, and environmental toxins, have a more established epigenetic connection, early-life stress (ELS) in the postnatal years is less studied but may be just as impactful in terms of phenotypic outcomes. A major intermediary between ELS and ASD is likely the hypothalamic–pituitary–adrenal axis (HPA axis), which has been shown to be epigenetically modified by ELS and whose genes and dysfunction overlap with ASD genes and symptoms. In this narrative review, we synthesize human and animal evidence to examine the relationships between ELS and ASD through epigenetic regulation of a non-exhaustive list of autism candidate genes involved in the HPA axis, including *NR3C1*, *FKBP5*, *MECP2*, *GAD1*, *RELN*, *SHANK3*, *OXTR*, and *BDNF*. We discuss how ELS-induced epigenetics may modulate HPA axis negative feedback, and how epigenetic alterations in this pathway and associated genes could affect ASD phenotypes.

## Simple Summary

Autism spectrum disorder is a complex condition whose causes are not fully understood, though both genetic and environmental factors play a role. This review explores how stressful experiences during early childhood, such as abuse, neglect, parental loss, or poverty, may contribute to autism through changes in how genes are turned on or off, a process called epigenetics. These changes do not alter the genetic code itself but can affect brain development and behavior. This review focuses specifically on genes involved in the body’s stress response system, which releases hormones like cortisol to help us cope with challenges. When this system becomes dysregulated, it can lead to chronic stress and developmental problems. By examining research from both human and animal studies, this review identifies several genes where early stress appears to cause epigenetic changes that overlap with genes that are already implicated in autism. This suggests that people already at risk for increased early-life stress and emotional dysregulation are subject to epigenetic changes that exacerbate the body’s physical response to stress. Understanding these connections is valuable because it may reveal biological markers that help identify at-risk children earlier and point toward new treatment approaches.

## 1. Introduction

Autism spectrum disorder (ASD) is a complex disorder with numerous causes that interact with each other to generate the wide range of symptoms observed across affected individuals. It is characterized by defects in social communication and interactions, and restrictive, repetitive patterns of behaviors and interests [1] (Takeda et al., 2024). Recent large-scale sequencing and meta-analysis studies now show that high-penetrance rare variants (de novo or inherited single-nucleotide polymorphisms, insertions, deletions, or copy-number changes) are identifiable in up to 30% of autism diagnoses, leaving about 70% of cases without a single, clear genetic association [2] (Costa et al., 2025). However, there is a growing interest in the effects of epigenetic mechanisms on the manifestation of and symptom severity in ASD.

Epigenetics is the regulation of gene expression through cell- and tissue-specific modifications such as DNA methylation, acetylation, and histone modifications, which do not change the DNA sequence directly, but rather determine which regions of genes are expressed. It has been shown that epigenetic changes are caused by environmental factors such as pollutants, stress, and nutrition [3] (Kuodza et al., 2024). While exposure to these factors during gestation has strong implications, it is also known that the first years after birth are a sensitive period for proper development, and exposure to environmental factors during this period can cause defects in neurodevelopment via epigenetic modifications [3] (Kuodza et al., 2024). In particular, early-life stress (ELS) during childhood—including exposure to experiences such as maternal depression or anxiety, child abuse or neglect, poverty, loss of a parent, and family conflict and violence—has been implicated in possibly triggering or exacerbating ASD [1] (Takeda et al., 2024). This is particularly important because individuals with ASD are twice as likely to report four or more ELS exposures than typically developing (TD) individuals, and those with ASD exposed to ELS are much more likely to have comorbid conditions than TD individuals exposed to ELS [4] (Hoover & Kaufman, 2018). While this mechanism is not fully understood, epigenetics likely serves as a bridge between ELS and ASD.

The hypothalamic–pituitary–adrenal (HPA) axis may be a major player in the relationship between ELS and ASD. This pathway mediates the body’s stress response through a cascade of stress hormones, which eventually lead to the release of glucocorticoids (e.g., cortisol) that induce physiological reactions and negative feedback mechanisms. By suppressing further stress hormone secretion from the hypothalamus and pituitary gland, this feedback reduces cortisol levels and restores homeostatic balance. These negative feedback mechanisms are crucial to the function of the HPA axis, and dysregulation of it can be caused by epigenetic changes, which lead to deleterious effects [5] (Zimmermann et al., 2016). Specifically, dysregulation of the HPA axis with DNA methylation has been observed in individuals with ASD, and epigenetic modifications due to ELS could be an explanatory mechanism. A meta-analysis found that peripheral cortisol levels tend to be elevated in children with ASD, possibly reflecting chronic stress or a primed stress axis. In addition, animal models support that environmental stressors causing HPA dysfunction can induce autism-like behaviors [6] (Gao et al., 2022). This implies that regulatory genes of the HPA axis may overlap with those implicated in ASD. A more detailed explanation of the molecular pathway of the HPA axis, necessary to understand the mechanism of these epigenetic changes in both ASD and ELS, is provided in Section 2.

There are two big questions that arise when studying how ELS shapes the epigenetic landscape of ASD relative to the HPA axis: (1) how might ELS contribute directly to the development of ASD; and (2) does ELS mediate the expression of certain phenotypes in those already affected? This review aims to analyze the pathways through which ELS may lead to epigenetic modifications, particularly within the HPA axis, and to discuss the implications for ASD pathophysiology. Through the novel examination of candidate ASD genes known to be implicated in both ELS and epigenetic regulation, we highlight potential diagnostic biological biomarkers and therapeutic targets.

### Search Strategy and Study Selection

A systematic review of PubMed and Google Scholar was performed, limited to peer-reviewed articles and using the following search term combinations: (“early-life stress” OR “childhood adversity” OR “maternal deprivation” OR “postnatal stress”) AND (“epigenetics” OR “DNA methylation” OR “histone modification” OR “hydroxymethylation”) AND (“autism” OR “autism spectrum disorder” OR “ASD”) AND (FKBP5 OR NR3C1 OR MECP2 OR RELN OR GAD1 OR SHANK3 OR OXTR OR BDNF OR “HPA axis” OR “hypothalamic–pituitary–adrenal”). In addition, ELS and epigenetic search terms were sometimes only combined with target genes, and ASD and epigenetic search terms were also combined with only target genes as individual searches. Zotero was used to cite sources. The eligibility criteria for the articles were as follows: studies that used human participants (any age) with ASD or typically developing controls, and/or non-human mammalian models; studies that documented postnatal early-life stress (ELS) or validated an experimental analog (e.g., maternal separation, limited bedding); primary data on epigenetic marks (DNA/histone) in brain or peripheral (blood or saliva) tissue and at least one target ASD-implicated gene. Original quantitative studies were included, while case reports (<3 subjects), editorials, and conference abstracts were excluded.

## 2. Epigenetics and the HPA Axis

**Figure 1:**
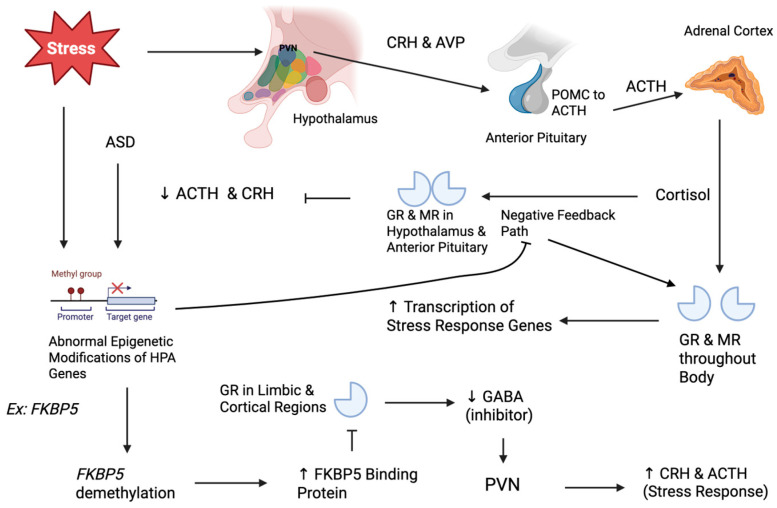
The HPA axis stress pathway and its epigenetic regulation. Solid black arrows indicate excitatory endocrine signaling along this cascade. Early-life stress (ELS) activates paraventricular nucleus (PVN) neurons in the hypothalamus to release corticotropin-releasing hormone (CRH) and arginine vasopressin (AVP) to the anterior pituitary, where they promote adrenocorticotropic hormone (ACTH) secretion. ACTH then stimulates glucocorticoid (cortisol) release from the adrenal cortex. Cortisol then acts on glucocorticoid receptors (GRs) and mineralocorticoid receptors (MRs) throughout the body, leading to increased transcription of stress response genes. Blunt-ended lines represent negative feedback. Transcriptional regulation direction is indicated by up and down arrows beside the effected protein. This is shown as eventually rising cortisol via GRs/MRs in the hypothalamus and anterior pituitary, which inhibits further CRH and ACTH release, regulating the stress response. FKBP5 demethylation is an epigenetic modification seen in both ASD and ELS. It leads to increased FKBP5 binding protein levels, increasing its negative feedback (blunted line) on GR in the limbic and cortical regions. This reduces GR sensitivity and GABAergic inhibition of the PVN, increasing CRH and ACTH release and contributing to dysregulation of the stress response.

To understand the effects of ELS, it is first important to understand the pathway of the HPA axis (Figure 1). It begins when stress causes the paraventricular nucleus (PVN) of the hypothalamus to release corticotropin-releasing hormone (CRH) and arginine vasopressin (AVP) [7] (Sheng et al., 2021). CRH and AVP induce the transcription of the pro-opiomelanocortin (*Pomc*) gene in the anterior pituitary gland, which is cleaved into adrenocorticotropic hormone (ACTH). ACTH travels through the blood to the adrenal glands, where it stimulates the synthesis and release of cortisol, which then regulates various physiological processes, including metabolism, immune function, and cardiovascular function. It does this by binding the mineralocorticoid receptor (MR) and the glucocorticoid receptor (GR). Both MR and GR are ligand-activated transcription factors. Once they bind to cortisol, the MR and GR complexes translocate from the cytoplasm into the nucleus and bind DNA sequences called hormone response elements (HREs) located in the promoter regions of target genes [7] (Sheng et al., 2021). These either enhance or suppress the transcription of stress response genes, such as those involved in cell survival, inflammation, and metabolism of glucose, lipid, and proteins. The rise in cortisol eventually leads to negative feedback when it binds to GR and GC receptors in the hypothalamus and anterior pituitary. This causes GR/MR complexes to translocate to the nucleus and bind glucocorticoid response elements in target genes, including those encoding CRH in PVN neurons and POMC in pituitary corticotropic cells. This genomic signaling recruits corepressors and reduces transcription of *Crh* and *Pomc*, inhibiting CRH and ACTH release [7] (Sheng et al., 2021). There are various stress-induced epigenetic changes proposed for genes encoding parts of the HPA axis or having a significant impact on it. These genes include *Fkbp5*, *Nr3c1*, *Mecp2*, *Gad1*, *Reln*, *Shank3*, *Oxtr*, and *Bdnf*, all of which have been implicated in ASD. Several of these are known as “syndromic” genes, in which rare, highly penetrant mutations cause distinct neurodevelopmental disorders. For example, loss-of-function *Mecp2* variants cause Rett syndrome, and *Shank3* deletions or truncating variants cause Phelan–McDermid syndrome. Here, we do not conflate these monogenic conditions with idiopathic ASD. Instead, we focus on whether more modest, environmentally shaped epigenetic alterations at the same loci—occurring in individuals without known pathogenic mutations—might influence ASD risk, symptom expression, or the response to early-life stress. These epigenetic changes occur through DNA methylation, which is the addition of methyl groups to nucleotide bases that either recruit transcription repressors/activators or repress transcription themselves. The common aspect of these pathways is dysregulation of the negative feedback mechanism of GR and GC receptors, or upregulation of components of the hormone cascade [8] (Weaver et al., 2004).

## 3. *FKBP5*

*Fkbp5* is a gene that encodes a binding protein for the GR, which decreases its responsiveness to cortisol. It is typically regulated by methylation that prevents its inhibitory effects on the negative feedback loop [9] (Klengel et al., 2013). However, it has been observed that early-life trauma can induce demethylation of the *FKBP5* gene, which increases its activity of inhibiting the GR and negative feedback mechanism, leading to a hyperactive HPA axis. Importantly, *FKBP5* epigenetic regulation has been shown to be linked to genetic predisposition for posttraumatic stress disorder (PTSD). In a study looking at the differences in DNA methylation levels of the *FKBP5* gene in groups that were samples of highly traumatized individuals from two larger trauma studies (the Grady and Conte trauma projects; [9] (Klengel et al., 2013), an average decrease of 12.3% in DNA methylation in intron 7 and two CpG regions was found. This decrease in methylation was correlated with a risk allele single-nucleotide polymorphism (SNP) for PTSD and was not observed in those with a protective genotype [9] (Klengel et al., 2013). An interesting observation in ASD is that a certain SNP within the *FKBP5* gene moderates the association between autistic traits and social anxiety [10] (Yang et al., 2021). This suggests a relationship between the dysregulation of FKBP5 in both stress and ASD, resulting in similar symptoms. Future research should examine whether this SNP influences epigenetic changes and exacerbation of ASD symptoms in a similar mechanism to PTSD, which would strengthen the relationship between the epigenetic effects of ELS and ASD. When treated with a GR agonist, Klengel and colleagues discovered a significant reduction in intron 7 DNA methylation of hippocampal neural progenitor cells in culture, which lasted through an incubation period of 20 days [9] (Klengel et al., 2013). However, this demethylation did not occur after cells had differentiated and proliferated [9] (Klengel et al., 2013). This suggests that the activation of GRs via ELS—which leads to DNA demethylation of the *FKBP5* gene—is particularly susceptible during childhood (when progenitor cells are most abundant) and is long-lasting. The result is GR resistance to cortisol and a lack of its negative feedback.

Further evidence of epigenetic dysregulation of *FKBP5* in ASD suggests an association between ELS and ASD. Postmortem brain studies have reported increased FKBP5 expression in individuals with ASD across regions such as the frontal cortex, hippocampus, and cerebellum [11] (Patel et al., 2016). One study found an estimated 42% FKBP5 mRNA increase in the middle frontal gyrus of patients with ASD compared to controls [11] (Patel et al., 2016). In the same ASD brains, GR levels were significantly reduced (see NR3C1 below), consistent with an impaired negative feedback loop caused by a lack of inhibitory signals from these regions to the hypothalamus (Figure 1). Therefore, excess FKBP5 in ASD may contribute to cortisol feedback resistance and atypical stress responses characteristic of a hyperactive HPA axis, noted in many individuals with ASD and those with a history of ELS [11] (Patel et al., 2016). Elevated FKBP5 in ASD could be due to genetic and/or environmental influences, including possible ELS exposure in some cases. In summary, ELS leads to a dysfunctional HPA feedback loop driven by FKBP5 cortisol dysregulation (high FKBP5 and low GR), which may play a mediating or exacerbating role in the anxiety, irritability, and stress-management dysregulation seen in individuals with ASD.

## 4. *NR3C1*

Another gene that has implications in both ELS and ASD is the *Nr3c1* glucocorticoid receptor gene; this has been shown to be hypermethylated following stress, which leads to its downregulation and diminished negative feedback regulation of the HPA axis. For example, in the famous Weaver et al. study, rats with mothers who had low licking/grooming (LG) behavior displayed increased methylation of CpG sites in the exon i7 promoter region of the *Nr3c1* gene [8] (Weaver et al., 2004). This was associated with an increased stress response, which, interestingly, could be reversed—as shown through cross-fostering experiments where biological offspring from high-LG mothers were raised with low-LG mothers and vice versa [8] (Weaver et al., 2004). They discovered that high-LG mothers reversed the high methylation levels due to low LG after care and returned to normal methylation levels. These results highlight the importance of the environment in improving a child’s development (e.g., through increased parental care), which has direct implications for ASD intervention strategies. Another important finding from the study was the effect of histone deacetylase (HDAC) inhibitor trichostatin A (TSA) on the methylation levels of low-LG mice. HDAC removes acetyl groups, preventing transcription factor binding and promoting DNA methylation. Treatment with TSA resulted in DNA demethylation in low-LG rats that initially had high levels of methylation, showing that ELS-induced epigenetic alterations in the HPA axis can have biochemical reversal pathways with therapeutic potential [8] (Weaver et al., 2004). In humans, the *NR3C1* gene has also been shown to be subject to increased methylation with prenatal maternal stress, which predicts HPA axis reactivity in offspring [12] (Kundakovic & Jaric, 2017). While postnatal ELS has not been examined directly, these findings support the idea that *NR3C1* is epigenetically susceptible to environmental stress, which leads to a dysfunctional HPA axis.

Importantly, these findings have been connected to ASD. For example, Oh et al. discovered that individuals with ASD had significantly higher DNA methylation of the *NR3C1* gene than controls, demonstrating a positive correlation between the effects of ELS and ASD [13] (Oh et al., 2024). This aligns with findings from Patel et al. [11], with postmortem analyses of the frontal cortex in individuals with ASD showing significantly lower NR3C1 mRNA and protein levels compared to controls [11] (Patel et al., 2016). They found that multiple GR isoforms (GRα, GRγ, and GR-P) were reduced by 20–64% in ASD brains, accompanied by a 46% decrease in mineralocorticoid receptor (MR) mRNA. The drop in GRα is especially important because it is the major functional isoform and would limit cortisol’s negative feedback efficacy. Notably, no changes in GRβ (a dominant-negative isoform) were found, suggesting the decrease in GR is not due to alternative splicing but likely reduced gene expression [11] (Patel et al., 2016). Overall, both ASD and ELS are characterized by an underactive GR system via DNA methylation, which leads to a hyperactive HPA axis. Future studies should investigate whether ELS plays a moderating or causative role in ASD.

## 5. *MECP2*

The *MECP2* gene codes for the MeCP2 protein, which binds to methylated CpG sites and recruits corepressors, decreasing gene expression. This gene has been shown to be dysregulated in ASD, with both over- and underexpression resulting in ASD symptoms [14] (Zhou et al., 2019). These defects can be a result of both genetic and epigenetic causes, with novel missense mutations in the gene, as well as increased promoter methylation being detected in individuals with ASD [15] (Nagarajan et al., 2006). While genetic mutations are not a direct result of epigenetic modifications, because MeCP2 modulates the epigenetic response to factors including ELS, mutations in patients with ASD could enhance the epigenetic impact of ELS in such patients. This provides a link between ELS and its increased effects in patients with ASD, although coding mutations are rarer than epigenetic changes [15] (Nagarajan et al., 2006). For example, gain-of-function mutations that increase MeCP2 expression have been found in patients with ASD, where increased MeCP2 binding to promoters of *GAD1* and *RELN* genes has been observed [16] (Zhubi et al., 2014). GAD1 is responsible for GABA synthesis, and RELN regulates synaptic plasticity; their dysregulation has been associated with ASD [17] (Zhubi et al., 2017). In the cerebral cortex of individuals with ASD, the positive correlation between MECP2 mRNA levels and binding to these genes could explain their dysregulation in ASD and exacerbation of epigenetic effects due to ELS. However, more directly, ASD is associated with epigenetic modifications of *MECP2*, which support a stronger relationship between ELS and ASD. It has been shown that increased *MECP2* promoter methylation occurs in the frontal lobes of males with ASD, which leads to decreased MeCP2 protein expression [15] (Nagarajan et al., 2006). This methylation is not associated with proximal genetic factors, in contrast to other neurological disorders like fragile X syndrome, where FMR1 promoter methylation is influenced by 5′ trinucleotide repeats [15] (Nagarajan et al., 2006). In addition, it does not appear to be due to irregular brain development because in individuals with Down Syndrome, who also express lower levels of MeCP2 protein, there was no abnormal methylation observed [15] (Nagarajan et al., 2006). These findings suggest that the *MECP2* methylation in individuals with ASD is unique and not due to innate genetic mechanisms of neurological disorders, but rather potentially to environmental factors.

To further elucidate the particular role of ELS in MeCP2 expression, it has been shown that ELS leads to increased neuronal activation. For example, maternal deprivation in mice reduces DNA methylation of the *Avp* gene [18] (Szyf, 2011). AVP induces CRH from the hypothalamus to release ACTH from the anterior pituitary, starting the stress hormone cascade. The proposed pathway is as follows: ELS causes HPA axis signaling, where hormones like ACTH and CRH act on receptors in neurons that stimulate excitatory neurotransmitters like glutamate to be released, causing depolarization. This excess depolarization leads to voltage-gated calcium influx and activation of Ca^2+^/calmodulin-dependent protein kinase II (CaMKII), which phosphorylates MeCP2 [18] (Szyf, 2011). Phosphorylated MeCP2 has decreased binding affinity for methylated *Avp*, leading to DNA demethylation of *Avp* [18] (Szyf, 2011). The upregulation of *Avp* via this ELS-induced epigenetic pathway then leads to hyperactivation of the HPA axis. The resulting abnormalities in stress response could also have implications for ASD, as both the symptoms of ELS and the resulting decrease in MeCP2 overlap with those observed in ASD [15] (Nagarajan et al., 2006). In addition, this specific pathway has a similar effect by demethylating *BDNF* [18] (Szyf, 2011). These are examples of a general proposed mechanism of inactivation of the *MECP2* gene due to overexcitation from ELS, leading to decreased inhibition of certain genes, which could explain the overexpression of genes also implicated in ASD.

## 6. *GAD1*

As previously discussed, the dysregulation of *MeCP2* due to ELS has an important impact on the expression of the *GAD1* gene. The *GAD1* gene has been further implicated as an epigenetic target in individuals with ASD, and could play a role in the relationship between ELS and ASD. It encodes glutamate decarboxylase 67 (GAD67), the enzyme responsible for synthesizing γ-aminobutyric acid (GABA)—the major inhibitory neurotransmitter of the brain [19] (Miyata et al., 2021). It has been shown to be reduced in the brains of individuals with ASD, and the lack of inhibition could explain excitatory symptoms of ASD like seizures, anxiety, and sensory hyper-responsivity [17] (Zhubi et al., 2017). The frontal cortex of individuals with ASD has been observed to have decreased *GAD1* mRNA levels due to increased MeCP2 binding at the promoter [17] (Zhubi et al., 2017). However, interestingly, this was not found to correlate with increased DNA methylation at the promoter, but with hydroxymethylation (5-hmC) [17] (Zhubi et al., 2017). 5-hmC is a derivative of methylation that leads to demethylation, serves as its own epigenetic mark that MeCP2 binds to, and has been implicated in the stress response [20] (Dick & Chen, 2021). These results are supported by findings showing an increase in the 5-hmC-to-5-mC ratio as well as MeCP2 binding in GAD1 in the cerebella of patients with ASD, which was correlated with a decrease in GAD1 expression [17] (Zhubi et al., 2017). This was associated with an increased expression of TET1 in ASD cerebella, which encodes the enzyme responsible for converting methylation into hydroxymethylation [17] (Zhubi et al., 2017). However, this change in 5-hmC from 5-mC was not due to MeCP2 and could rather be due to exposure to stress like ELS because ELS has been associated with increased TET1 activity [21] (Cheng et al., 2018), providing another possible link between ELS and ASD. This is similar to the results from another study, which showed that decreased DNA methylation in cerebral organoids of individuals with ASD led to increased binding of the CTCF transcriptional repressor and increased suppression of the *GAD1* gene [22] (Pearson et al., 2022). While the details of these mechanisms are different, they suggest a complex effect of differential methylation on the expression of certain ASD genes due to stress. These different pathways and nuances seem to converge to the common end result of decreasing *GAD1* expression and GABA levels in ASD and ELS-exposed individuals, disrupting the homeostasis of excitation and inhibition in the brain.

## 7. *RELN*

The *RELN* gene encodes the Reelin protein, which aids in neuronal migration and strengthening of synapses during brain development. It has also been found to be a target of MECP2 binding to its promoter, which is thought to repress its expression [17] (Zhubi et al., 2017). In addition, it has been shown to be differentially methylated in individuals with ASD. For example, in postmortem temporal cortical tissue, it was found that while overall methylation percentage in the *RELN* promoter was similar between ASD and controls, the pattern of methylation was distinct [23] (Lintas et al., 2016). ASD brains had significantly heavier methylation at specific CpG sites in the 5′ regulatory region of *RELN* (upstream of the transcription start site), which was different from the pattern of methylation in controls. *RELN* has also been shown to be regulated by epigenetic changes due to stress. In animal models, it was found that when mouse mothers experienced stress during gestation, this prenatal stress led to a significant increase in methylation of the *RELN* promoter in the cerebral cortex of the offspring and fewer RELN-expressing neurons [24] (Palacios-García et al., 2015). These epigenetic marks were stable throughout life and were correlated with behavioral phenotypes of increased anxiety, hyperactivity, and memory impairments, which are also associated with ASD, supporting the link between ELS and ASD [24] (Palacios-García et al., 2015). There is less literature on the impacts of early-life stress, but one article did reveal an interesting finding that RELN expression increased in rats subjected to maternal deprivation through decreased methylation of *Reln* in the hippocampus [25] (Wang et al., 2018). This is proposed to lead to strengthening of fearful memories and increased stressful responses throughout life. These findings could be relevant to individuals with ASD as they show that different impacts on RELN expression in ASD and ELS could lead to an end result of exacerbated ASD symptoms and comorbidities like increased hyperactivity.

The apparent contradictions between ELS and ASD epigenetic effects on *Reln* warrant further investigation as they reveal potential mechanisms behind the diversity of ASD symptoms. They show that the relationship between the epigenetic impacts of ELS and ASD is more nuanced and not as straightforward as a positive correlation in every gene. Rather than a contradiction, this could be because of differences in the brain regions studied. In the rat studies showing *Reln* hypomethylation, researchers assayed the hippocampus CA1 region [25] (Wang et al., 2018), a region involved in stress responses and memory. In contrast, the autism studies showing hypermethylation focused on cortical regions (including the cerebral cortex) and cerebellum [23] (Lintas et al., 2016). A mouse study also showed hypermethylation in the cerebral cortex due to prenatal stress [24] (Palacios-García et al., 2015). Therefore, perhaps ELS induces hypomethylation of *Reln* specifically in the hippocampus to increase the response to future stress, but induces hypermethylation in the cerebral cortex, where ELS might instead reduce connectivity. ASD primarily involves disruptions in the circuits of the cortex and cerebellum, while the hippocampus in ASD has been shown in some studies to contribute to hypersensitivity when presented with aversive stimuli [26] (Banker et al., 2021). Therefore, *Reln* hypermethylation in ASD cortices might be consistent with the repressive impact of ELS in that region, while the hippocampal hypomethylation seen in some rodent ELS models may be consistent with an excitatory mechanism for another phenotype of ASD. This reflects how different mechanisms due to ELS could contribute to the heterogeneity of symptoms in ASD.

## 8. *SHANK3*

*Shank3* is another gene that encodes a protein that supports synaptic connections, specifically through the development and maturation of dendritic spines. It has also been shown to undergo epigenetic regulation in both ASD and ELS. A study by Zhu et al. [27] discovered that three CpG islands in the *Shank3* gene had significantly increased methylation in the postmortem brains of individuals with ASD relative to controls [27] (Zhu et al. 2014). About 15% of ASD cases had clusters of hypermethylation at two of the three specific CpG islands, which corresponded with altered SHANK3 mRNA splicing and reduced levels of certain SHANK3 isoforms in these individuals. They also confirmed the causal relationship between methylation and altered gene expression by exposing cultured cells to a DNA methylation inhibitor in vitro, which reversed the methylation pattern at *shank3* and restored normal SHANK3 expression. They noted that this epigenetic modification indicates *shank3* might be particularly susceptible to environmental influences, and proposed *shank3* hypermethylation as a biomarker of gene–environment interactions in ASD. Continuing this line of reasoning, subsequent research in animals has shown that environmental exposures can have an impact on *shank3* epigenetics. For example, Li et al. [28] found that infant rats exposed to high levels of environmental pollution (fine particulate matter in air) developed autism-like phenotypes (social deficits and repetitive behaviors), had increased methylation of the *shank3* promoter in the frontal cortex, and reduced SHANK3 expression [28] (Li et al., 2023). Although direct evidence of ELS affecting *shank3* methylation is limited, this finding aligns with the mechanism of *shank3* being epigenetically susceptible to adverse environmental conditions, which can weaken synaptic scaffolding and plasticity. Therefore, it is possible that ELS induces similar *shank3* methylation changes as it does in other genes during the critical period of development, resulting in the abnormalities in neural circuits for social communication also commonly found in ASD.

## 9. *OXTR*

The hormone oxytocin plays an important role in both social bonding and stress regulation, which are often affected in ASD [29] (Loke et al., 2015). Defects in *OXTR*, the gene coding for the oxytocin receptor, have been linked with ASD, with certain SNPs correlating with social function deficits and repetitive behaviors [29] (Loke et al., 2015). Importantly, the gene has also been shown to be epigenetically impacted in ASD [30] (Gregory et al., 2009), with a study finding significantly increased DNA methylation at the *oxtr* promoter CpG island in a male with autism, and lower OXTR mRNA levels in the temporal cortex [31] (Kumsta et al., 2013). In animal models, ELS has also been shown to be correlated with hypermethylation of *oxtr*. For example, prairie vole pups subjected to low maternal care showed de novo DNA methylation at key regulatory CpGs in *oxtr* within the nucleus accumbens, leading to reduced Oxtr expression and altered protein distribution [32] (Perkeybile et al., 2019). It was also found that *oxtr* methylation changes in blood were correlated with the same changes in the brain, highlighting a systemic effect of ELS and supporting the reliability of blood tests for *oxtr* methylation [32] (Perkeybile et al., 2019). Similarly, in humans, adults who reported low maternal caregiving in their childhood had significantly higher *oxtr* methylation in their blood cells compared to those reporting high levels of care [33] (Unternaehrer et al., 2015). Another human study also reported that less-nurturing maternal care was associated with increased *oxtr* methylation in offspring [34] (Simons et al. 2017). Overall, ELS seems to be associated with increased *oxtr* promoter methylation in both the blood and brain, which correlates with the methylation trends seen in ASD as well. This suggests that ELS could be a moderator of ASD symptoms. For example, if a child with genetic vulnerability due to ASD is also exposed to ELS, the added epigenetic suppression of *oxtr* could exacerbate social impairment. Coming back to the HPA axis and its dysregulation in ASD, the *oxtr* gene could help explain the impacts of the HPA axis on ASD symptoms due to ELS [35,36] (Lancaster et al., 2018; Puglia et al., 2015). *Oxtr* methylation might be why some individuals with ASD have greater anxiety or stress reactivity, because a repressed oxytocin system due to methylation from ELS could, in turn, fail to properly regulate HPA axis overactivation caused by ELS [35,37,38] (Lancaster et al., 2018; Wang et al., 2021; Puglia et al., 2018). However, further studies are needed to establish any causality between ELS and ASD through *oxtr* methylation.

## 10. *BDNF*

Finally, the *BDNF* gene codes for brain-derived neurotrophic factor (BDNF), which, like many other ASD-implicated genes, modulates synaptic strength, plasticity, and circuit formation [39] (Porcher et al., 2018). It also regulates excitatory/inhibitory homeostasis in the brain by upregulating presynaptic GABA synthesis and supporting inhibitory synapse formation [39] (Porcher et al., 2018). It has been implicated in ASD, with several studies reporting that individuals with autism have dysregulated BDNF levels in the brain and blood, although results have been mixed. For example, both abnormal increases and decreases in expression of BDNF mRNA and protein in cortical tissue from patients with ASD have been observed [39] (Porcher et al., 2018). Specifically, it was found that BDNF expression is increased in clinically mild ASD cases but decreased in clinically severe (typical ASD) cases [40] (Kasarpalkar et al., 2014). This suggests that the increases could be a potential compensatory response in individuals with ASD whose BDNF is still functional enough to enact a protective response to the abnormal connectivity in ASD. In addition, results from animal models have supported a more direct relationship. For example, male mice engineered with lower activity-dependent BDNF release showed significant social avoidance and increased self-grooming, a repetitive behavior representative of ASD, supporting the mechanism that lower BDNF contributes to key ASD symptoms [41] (Ma et al., 2023).

ELS has also shown a consistent reduction in BDNF expression through hypermethylation in both animal and human models. For example, infant rat pups exposed to abusive caretaking or repeated maternal separation had significantly lower BDNF mRNA in the prefrontal cortex as adults, as well as hypermethylation of the *bdnf* exon IV promoter in that region [42] (Roth & Sweatt, 2011). Importantly, experimentally reversing DNA methylation with a demethylating drug restored BDNF levels in the previously stressed rats [42] (Roth & Sweatt, 2011), which confirmed that DNA methylation was repressing BDNF. In humans, other studies have similarly reported that children subject to ELS, such as childhood abuse and low maternal care, have elevated *bdnf* promoter methylation in peripheral blood or brain tissue [33] (Unternaehrer et al., 2015). This hypermethylation of *bdnf* has also been shown to impact the HPA axis, as rodent experiments using maternal separation showed reduced hippocampal BDNF and synaptogenesis at baseline, which correlated with symptoms like greater anxiety, less social interaction, and HPA hyperactivity [43] (Daskalakis et al., 2015). Therefore, it seems that ELS decreases BDNF signaling through epigenetic suppression, weakening neural support for stress regulation. Besides the inconsistency in BDNF expression in mild ASD, alterations to BDNF in ELS and typical ASD result in similar dysfunctions in neurodevelopment and synaptic plasticity, which suggests ELS might exacerbate ASD phenotypes. Furthermore, environmental factors have been correlated to changes in BDNF in ASD, as studies have shown that children with autism who experienced adversity had differences in *bdnf* DNA methylation, suggesting a convergence of ELS and autism on *bdnf* regulation [44] (Forsberg et al., 2018). In addition, since reduced BDNF results in fewer or immature GABAergic interneuron development, hypermethylation due to ELS could result in hyperexcitation in the brain, which is also observed in ASD, with its excitation-over-inhibition imbalance that manifests in symptoms such as seizures [39] (Porcher et al., 2018). Epigenetic reduction in BDNF due to ELS could also be related to the abnormal cortical connectivity in frontal and hippocampal circuits, which results in learning deficits, memory impairments, and increased anxiety—observed in both children who experience ELS and those with ASD [39] (Porcher et al., 2018). Therefore, in addition to a more causal role, ELS could also play a moderating role in children with ASD by further reducing BDNF in key brain regions, exacerbating ASD symptoms such as learning difficulties and inflexible thinking. In summary, the convergence of ELS and ASD on *bdnf* methylation implies that dysfunctional BDNF lowers support for neuronal development during critical periods by disrupting GABAergic signaling, synaptic plasticity, and cortical development [39] (Porcher et al., 2018). This may also play a role in the manifestation of ASD symptoms, revealing potential relationships between ELS and ASD through BDNF.

## 11. Discussion

Early-life stress is associated with lasting epigenetic changes, particularly in genes key to the HPA axis function. The genes discussed here each play a role in modulating stress responses and neural development. In a normally functioning HPA axis, cortisol elevation triggers GR-mediated feedback that limits further cortisol release; oxytocin signaling dampens amygdala reactivity and promotes social buffering; BDNF provides neurotrophic support to maintain healthy feedback circuitry; and MeCP2 regulates stress-excitatory genes. However, their dysregulation due to ELS disrupts this balance and leads to a common result of HPA axis hyperactivity. Similar dysregulations are often found in ASD, potentially causing or contributing to the high comorbidity of stress-related issues in autism [44] (Forsberg et al., 2018). Across studies, ELS is associated with promoter hypermethylation of *nr3c1*, *oxtr*, and bdnf and hypomethylation within *fkbp5* GREs. ASD tissues frequently show similarly reduced GR expression and altered FKBP5, GAD1, RELN, SHANK3, OXTR, and BDNF signaling. These marks cluster at nodes that regulate glucocorticoid feedback, inhibitory neurotransmission, and social-stress circuitry. For example, hypermethylation of *nr3c1* and demethylation of *fkbp5* due to ELS result in inadequate negative feedback and persistent cortisol secretion [45] (Jones, 2013). Likewise, BDNF loss and *oxtr* silencing degrade the neural and hormonal buffers against stress, leading to overactivation [33,42] (Roth & Sweatt, 2011; Unternaehrer et al., 2015). In ASD, possibly due to environmental stress such as ELS, there is often a comparable pattern of HPA axis dysregulation symptoms (e.g., elevated cortisol and atypical stress reactivity). These reflect the molecular irregularities such as low GR and high FKBP5 found in the brain and blood [11] (Patel et al., 2016). Crucially, these molecular changes are reflected in real-world behaviors. Reduced GR and elevated FKBP5 result in a child who struggles to calm down after stress exposure and appears chronically anxious or hyperactive. Low OXTR and low BDNF can manifest in social withdrawal, poor attachment, and heightened anxiety since the protective effects of social interaction and neural plasticity for adaptation are reduced [37,39] (Porcher et al., 2018; Wang et al., 2021). Dysregulated MeCP2 may broadly amplify neural noise and stress sensitivity, contributing to autism’s sensory overload and anxiety [36] (Puglia et al., 2015).

The brain regions involved with these genes also support these mechanisms. The hippocampus and prefrontal cortex are important for inhibitory control over the HPA axis and executive function, and their dysfunction due to impairment of these genes leads to both hormonal and cognitive dysregulation [42] (Roth & Sweatt, 2011). The amygdala is rich in OXTR and BDNF, and when deprived of their support becomes hyperreactive, promoting fear and stress responses [46] (Hill et al., 2014). If the hypothalamus receives inadequate inhibition and excess AVP from low MeCP2, it would oversecrete CRH [18] (Szyf, 2011). Meanwhile, the anterior pituitary, possibly freed from *mecp2* repression, secretes more ACTH, and, facing less negative feedback due to less GR activity, keeps stimulating the adrenals [11,18] (Patel et al. 2016; Szyf, 2011). This paints a picture of a hyperactive HPA axis under allostatic load, the wear-and-tear of chronic stress hormone exposure [47] (Kinlein et al., 2015). Over time, this allostatic load can further establish detrimental cycles of neural changes. For example, cortisol toxicity can erode hippocampal dendrites [48] (Liston & Gan, 2011), reducing GR expression even more, and chronic low oxytocin can impede social learning. These feedback loops could be particularly detrimental in ASD development because these pathways are already dysregulated and could increase the severity of the disorder by amplifying core ASD phenotypes.

**Figure 2:**
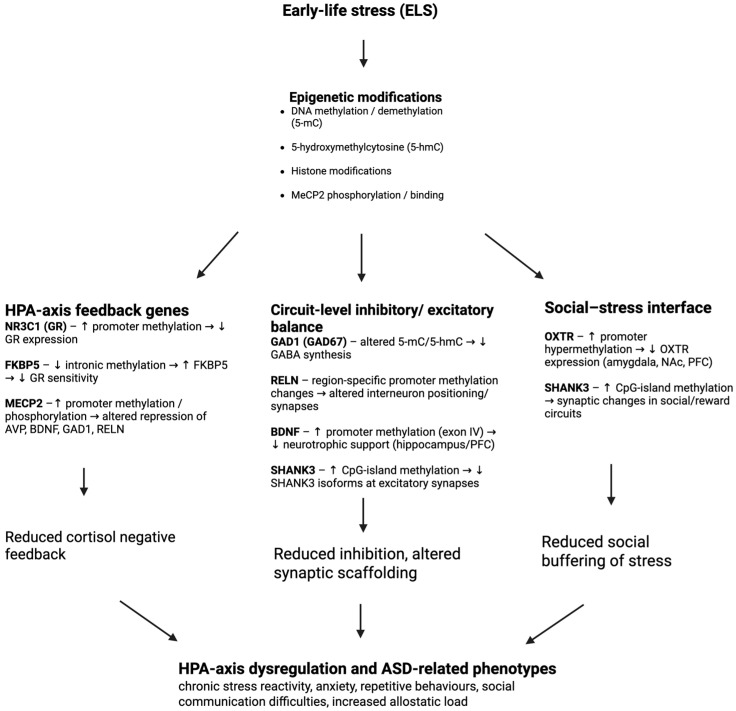
Epigenetically regulated genes linking early-life stress, the HPA axis, and ASD. Transcriptional regulation direction is indicated by up and down arrows beside the effected protein.

The genes discussed can be summarized into three partially overlapping functional groups (Figure 2): NR3C1 and FKBP5 serve as HPA axis feedback regulators; MECP2, GAD1, RELN, BDNF, and SHANK3 are epigenetic and interneuron regulators that shape inhibitory/excitatory balance and synaptic development; OXTR and SHANK3 support social-bonding and salience systems that modulate the stress response [39,49] (Porcher et al., 2018; Makris et al., 2021). Transcriptional regulation direction is indicated by up and down arrows beside the effected protein. ELS-related epigenetic alterations (*nr3c1* promoter hypermethylation, *fkbp5* demethylation, *mecp2* phosphorylation or promoter methylation, increased 5-hmC at *gad1* and *reln*, *shank3* hypermethylation, and *oxtr* and *bdnf* promoter hypermethylation) push these groups toward a state of chronic hyper-responsivity to stress and reduced capacity for neuroplasticity [8,16,17,42] (Weaver et al., 2004; Zhubi et al., 2014, 2017; Roth & Sweatt, 2011). Importantly, many of the same genes are independently altered in ASD cohorts, suggesting that ASD and ELS frequently impact overlapping, rather than entirely separate, epigenetic pathways [11,30,44] (Gregory et al., 2009; Patel et al., 2016; Forsberg et al., 2018).

While the current literature does not support a causal link between ELS and ASD, recognizing these connections has implications for future, more targeted interventions. For example, therapies that could demethylate key promoters or boost receptor expression—such as GR or OXTR agonists, or HDAC inhibitors (as in the Nr3c1 rat)—have the potential to restore more normal HPA axis function. Likewise, behavioral/social interventions specifically targeted to ASD or at-risk individuals that provide a high nurturing environment could potentially reverse ELS-induced epigenetic marks, as seen in cross-fostering in animals. The interplay between ELS and ASD, shown through these genes, as well as the increased risk of ELS exposure for individuals with ASD, provides insight for physicians to include ELS as a major factor to consider for preventing, diagnosing, and treating patients with ASD. Therefore, understanding the nuances of this relationship provides hope that we can prevent at-risk children from the long-term impacts of early-life stress and perhaps improve stress resilience in those with ASD by repairing the molecular feedback loops of the HPA axis.

## 12. Conclusions and Future Directions

While the available evidence for ELS’s epigenetic impact on ASD-related mechanisms is encouraging, several knowledge gaps highlight promising avenues for future research. Many of the human studies are correlational and do not reveal whether the epigenetic changes caused the ASD traits or if a third factor, such as genetic predisposition, caused both ASD and methylation changes. Prospective longitudinal studies from birth through childhood with epigenetic sampling pre- and post-ELS are needed to establish causality. The results of these studies have the potential to significantly strengthen our understanding of the temporal relationship between stress exposure, epigenetic modifications, and the emergence of ASD phenotypes.

The variability in stressful experiences across studies presents another challenge for the field, as the epigenetic effects may differ by stressor type, timing, genetic susceptibility, and severity. Many studies group diverse adversities together, which has the potential to confound results. Future research should aim to differentiate these variables to identify specific epigenetic signatures associated with particular forms of early adversity. Individual genetic profiles and other biological factors could also result in differential susceptibility to epigenetic effects. In addition, much of the human epigenetic data came from peripheral tissues (blood or saliva), which may not reflect brain-specific changes, and even within the brain, there are region-specific epigenetic patterns among the same genes [50] (Gutierrez-Arcelus et al., 2015). Developing reliable peripheral biomarkers that correlate with neural processes represents a crucial next step for translational research. Cross-disciplinary approaches integrating neuroimaging techniques with epigenetic analyses could help bridge this gap and enhance our understanding of how peripheral markers relate to central nervous system function in individuals with ASD.

With regard to differential gene expression, males and females might epigenetically respond differently to ELS, and ASD has a strong male bias. Some studies found that *oxtr* methylation changes are more pronounced in females with trauma, yet ASD is less common in females, who perhaps require a higher burden of “hits” (environmental and genetic influences) for expression [51] (Schaafsma, et al. 2017). Understanding sex-specific epigenetic resilience mechanisms represents an important frontier for research that could explain differential vulnerability to ASD and inform sex-specific interventions.

Finally, the present review focuses primarily on DNA methylation/5-hmC and chromatin-based mechanisms at HPA axis-related genes and does not systematically cover non-coding RNA (ncRNA) regulation. The growing literature indicates that non-coding RNAs can also modulate HPA axis components and ASD-implicated pathways, and ncRNA-mediated mechanisms may interact with the DNA methylation changes discussed here. A comprehensive synthesis of ncRNA findings in ELS and ASD is beyond the scope of this article but represents an important direction for future work.

The convergence of stress-related and ASD-implicated genetic pathways identified in this review (summarized in Table 1) underscores the importance of examining gene–environment interactions through an epigenetic lens. *Fkbp5*, *nr3c1*, *mecp2*, *reln*, *gad1*, *oxtr*, and *bdnf* each uniquely illustrate how epigenetic regulation of stress-related genes links ELS to ASD through interconnected pathways of HPA negative feedback, circuit-level excitation/inhibition balance, and social buffering of stress (Figure 2). They potentially explain why individuals with ASD who experience ELS often show amplified anxiety, stress reactivity, and ASD symptoms. This is likely because ELS could exacerbate dysfunction in both stress-regulating and ASD-implicated genes, which overlap and may already be dysregulated. Future research exploring these intersections may yield insights into personalized interventions targeting epigenetic modifications during critical developmental windows, potentially mitigating the compounding effects of early adversity on ASD symptomatology. Multi-gene epigenetic “stress signatures” incorporating *nr3c1*, *fkbp5*, *oxtr*, and *bdnf* could, with sufficient validation, serve as biomarkers for identifying youth with autism who are particularly vulnerable to ELS-related symptom exacerbation [35,37,44] (Forsberg et al., 2018; Lancaster et al., 2018; Wang et al., 2021). Experimental work in animals already demonstrates that some ELS-induced marks on HPA axis genes and BDNF are reversible with enriched caregiving, pharmacological modifiers of chromatin state, or targeted manipulation of GR or oxytocin signaling [8,32,42] (Weaver et al., 2004; Roth & Sweatt, 2011; Perkeybile et al., 2019). Together, these findings suggest an integrated framework in which psychosocial interventions (e.g., intensive caregiver support or stress-management training) and, eventually, carefully selected pharmacological agents are evaluated for their ability not only to improve behavior but also to normalize epigenetic regulation of HPA axis genes. Such a framework could reframe ELS-related epigenetic changes not as unchangeable risk markers, but as dynamic molecular displays of stress that may be resolved with early, personalized intervention. However, addressing the methodological challenges outlined above is essential to establishing a more causal link between ELS-induced epigenetic changes and ASD phenotypes and to translating these findings into effective clinical approaches.

## Figures and Tables

**Table 1:** Summary of epigenetic changes in each target gene in ASD and after ELS.

Gene	Function	Direction of Epigenetic Modifications After ELS (Human and Mammal Models)	Direction in ASD Tissue (Brain and Peripheral)	References
***NR3C1*** Glucocorticoid receptor	Ligand-activated nuclear receptor that mediates glucocorticoid negative feedback onto the HPA axis	Increased promoter methylation (mC) in mice	Increased promoter mC in humans	[8,13] Weaver et al. 2004; Oh et al. 2024
***FKBP5*** GR co-chaperone	Decreases GR ligand affinity and nuclear translocation; expressed in hippocampus, cortex, pituitary	Decreased intron 7 mC (human)	Decreased intron 7 mC (human)	[9,11] Klengel et al. 2013; Patel et al. 2016
***MECP2*** Methyl-CpG binding protein	Epigenetic reader in cortex and hypothalamus; binds methylated and hydroxymethylated CpGs and regulates transcription of AVP, BDNF, GAD1, RELN, and other stress-related genes	Increased phosphorylation (mice): decreased function	Increased promoter mC or coding variants; bidirectional expression changes leading to similar phenotypes	[15,18] Szyf et al. 2011; Nagarajan et al. 2006
***RELN*** Reelin	Organizes cortical/hippocampal synaptic plasticity, influencing cortical–hippocampal control of the HPA axis	Region-specific (humans): increased mC (cortex)/decreased mC (hippocampus)	Increased mC (mice cortex)	[23,24] Lintas et al. 2016; Palacios-García et al. 2015
***GAD1*** GAD67; glutamate decarboxylase 1	GABA synthesis: critical for inhibitory control of prefrontal, hippocampal, and cerebellar circuits that regulate stress responses	Increased hydroxymethylation (5-hmC), mixed mC (human)	Increased 5-hmC (human)	[17] Zhubi et al. 2017
***SHANK3*** *SH3*	Postsynaptic density scaffolding protein at glutamatergic synapses; shapes cortico-striatal and limbic circuits relevant to social behavior and stress coping	Limited data: increased promoter mC after pollution stress (rat)	Increased CpG-island mC; altered mRNA splicing (human)	[27,28] Li et al. 2023; Zhu et al. 2014
***OXTR*** Oxytocin receptor	G-protein-coupled receptor in hypothalamus, amygdala, and PFC; modulates social bonding and inhibition of HPA activation	Increased promoter mC (prairie voles)	Increased promoter mC (human)	[31,32] Perkeybile et al. 2019; Kumsta et al. 2013
***BDNF*** Brain-derived neurotrophic factor	Supports synaptogenesis, particularly of GABAergic interneurons, and hippocampal/prefrontal plasticity important for HPA feedback	Increased exon IV mC (rat); increased promoter mC (human)	Mixed: increased mC in mild ASD, decreased in severe ASD (human)	[33,40,42,44] Roth & Sweatt 2011; Unternaehrer et al. 2015; Kasarpalkar et al. 2014; Forsberg et al. 2018

## Data Availability

No new data were generated in the development of this review. Data sharing is not applicable to this article.

## References

[1] Takeda T., Makinodan M., Toritsuka M., Iwata N. (2024). Impacts of adverse childhood experiences on individuals with autism spectrum disorder. Curr. Opin. Neurobiol..

[2] Costa C.I.S., Madanelo L., Wang J.Y.T., da Silva Campos G., De Sanctis Girardi A.C., Scliar M., Monfardini F., de Cássia Mingroni Pavanello R., Cória V.R., Vibranovski M.D. (2025). Understanding rare variant contributions to autism: Lessons from dystrophin-deficient model. NPJ Genom. Med..

[3] Kuodza G.E., Kawai R., LaSalle J.M. (2024). Intercontinental insights into autism spectrum disorder: A synthesis of environmental influences and DNA methylation. Environ. Epigenetics.

[4] Hoover D.W., Kaufman J. (2018). Adverse Childhood Experiences in Children with Autism Spectrum Disorder. Curr. Opin. Psychiatry.

[5] Zimmermann C.A., Raabe F., Hoffmann A., Spengler D., Binder E. (2016). Epigenetic Programming of the HPA Axis by Early Life Adversity. Epigenetics and Neuroendocrinology: Clinical Focus on Psychiatry.

[6] Gao J., Zou J., Yang L., Zhao J., Wang L., Liu T., Fan X. (2022). Alteration of peripheral cortisol and autism spectrum disorder: A meta-analysis. Front. Psychiatry.

[7] Sheng J.A., Bales N.J., Myers S.A., Bautista A.I., Roueinfar M., Hale T.M., Handa R.J. (2021). The Hypothalamic-Pituitary-Adrenal Axis: Development, Programming Actions of Hormones, and Maternal-Fetal Interactions. Front. Behav. Neurosci..

[8] Weaver I.C.G., Cervoni N., Champagne F.A., D’Alessio A.C., Sharma S., Seckl J.R., Dymov S., Szyf M., Meaney M.J. (2004). Epigenetic programming by maternal behavior. Nat. Neurosci..

[9] Klengel T., Mehta D., Anacker C., Rex-Haffner M., Pruessner J.C., Pariante C.M., Pace T.W.W., Mercer K.B., Mayberg H.S., Bradley B. (2013). Allele-specific FKBP5 DNA demethylation mediates gene–childhood trauma interactions. Nat. Neurosci..

[10] OhYang T., Liu J., Zhang Y., Zhang Q., Shangguan L., Li Z., Luo X., Gong J. (2021). Coping style predicts sense of security and mediates the relationship between autistic traits and social anxiety: Moderation by a polymorphism of the FKBP5 gene. Behav. Brain Res..

[11] Patel N., Crider A., Pandya C.D., Ahmed A.O., Pillai A. (2016). Altered mRNA Levels of Glucocorticoid Receptor, Mineralocorticoid Receptor, and Co-Chaperones (FKBP5 and PTGES3) in the Middle Frontal Gyrus of Autism Spectrum Disorder Subjects. Mol. Neurobiol..

[12] Kundakovic M., Jaric I. (2017). The Epigenetic Link between Prenatal Adverse Environments and Neurodevelopmental Disorders. Genes.

[13] Oh M., Yoon N.-H., Kim S.A., Yoo H.J. (2024). Epigenetic Insights into Autism Spectrum Disorder: DNA Methylation Levels of NR3C1, ASCL1, and FOXO3 in Korean Autism Spectrum Disorder Sibling Pairs. Clin. Psychopharmacol. Neurosci..

[14] Zhou C., Yan S., Qian S., Wang Z., Shi Z., Xiong Y., Zhou Y. (2019). Atypical Response Properties of the Auditory Cortex of Awake MECP2-Overexpressing Mice. Front. Neurosci..

[15] Nagarajan R., Hogart A., Gwye Y., Martin M.R., LaSalle J.M. (2006). Reduced MeCP2 Expression is Frequent in Autism Frontal Cortex and Correlates with Aberrant MECP2 Promoter Methylation. Epigenetics.

[16] Zhubi A., Chen Y., Dong E., Cook E.H., Guidotti A., Grayson D.R. (2014). Increased binding of MeCP2 to the GAD1 and RELN promoters may be mediated by an enrichment of 5-hmC in autism spectrum disorder (ASD) cerebellum. Transl. Psychiatry.

[17] Zhubi A., Chen Y., Guidotti A., Grayson D. (2017). Epigenetic regulation of RELN and GAD1 in the frontal cortex (FC) of autism spectrum disorder (ASD) subjects. Int. J. Dev. Neurosci. Off. J. Int. Soc. Dev. Neurosci..

[18] Szyf M. (2011). DNA methylation, the early-life social environment and behavioral disorders. J. Neurodev. Disord..

[19] Miyata S., Kakizaki T., Fujihara K., Obinata H., Hirano T., Nakai J., Tanaka M., Itohara S., Watanabe M., Tanaka K.F. (2021). Global knockdown of glutamate decarboxylase 67 elicits emotional abnormality in mice. Mol. Brain.

[20] Dick A., Chen A. (2021). The role of TET proteins in stress-induced neuroepigenetic and behavioural adaptations. Neurobiol. Stress.

[21] Cheng Y., Sun M., Chen L., Li Y., Lin L., Yao B., Li Z., Wang Z., Chen J., Miao Z. (2018). Ten-Eleven Translocation Proteins Modulate the Response to Environmental Stress in Mice. Cell Rep..

[22] Pearson G., Song C., Hohmann S., Prokhorova T., Sheldrick-Michel T.M., Knöpfel T. (2022). DNA Methylation Profiles of GAD1 in Human Cerebral Organoids of Autism Indicate Disrupted Epigenetic Regulation during Early Development. Int. J. Mol. Sci..

[23] Lintas C., Sacco R., Persico A.M. (2016). Differential methylation at the RELN gene promoter in temporal cortex from autistic and typically developing post-puberal subjects. J. Neurodev. Disord..

[24] Palacios-García I., Lara-Vásquez A., Montiel J.F., Díaz-Véliz G.F., Sepúlveda H., Utreras E., Montecino M., González-Billault C., Aboitiz F. (2015). Prenatal Stress Down-Regulates Reelin Expression by Methylation of Its Promoter and Induces Adult Behavioral Impairments in Rats. PLoS ONE.

[25] Wang R.-H., Chen Y.-F., Chen S., Hao B., Xue L., Wang X.-G., Shi Y.-W., Zhao H. (2018). Maternal Deprivation Enhances Contextual Fear Memory via Epigenetically Programming Second-Hit Stress-Induced Reelin Expression in Adult Rats. Int. J. Neuropsychopharmacol..

[26] Banker S.M., Gu X., Schiller D., Foss-Feig J.H. (2021). Hippocampal contributions to social and cognitive deficits in autism spectrum disorder. Trends Neurosci..

[27] Zhu L., Wang X., Li X.-L., Towers A., Cao X., Wang P., Bowman R., Yang H., Goldstein J., Li Y.-J. (2014). Epigenetic dysregulation of SHANK3 in brain tissues from individuals with autism spectrum disorders. Hum. Mol. Genet..

[28] Li K., Liang X., Xie X., Tian L., Yan J., Lin B., Liu H., Lai W., Liu X., Xi Z. (2023). Role of SHANK3 in concentrated ambient PM2. 5 exposure induced autism-like phenotype. Heliyon.

[29] Loke Y.J., Hannan A.J., Craig J.M. (2015). The Role of Epigenetic Change in Autism Spectrum Disorders. Front. Neurol..

[30] Gregory S.G., Connelly J.J., Towers A.J., Johnson J., Biscocho D., Markunas C.A., Lintas C., Abramson R.K., Wright H.H., Ellis P. (2009). Genomic and epigenetic evidence for oxytocin receptor deficiency in autism. BMC Med..

[31] Kumsta R., Hummel E., Chen F.S., Heinrichs M. (2013). Epigenetic regulation of the oxytocin receptor gene: Implications for behavioral neuroscience. Front. Neurosci..

[32] Perkeybile A.M., Carter C.S., Wroblewski K.L., Puglia M.H., Kenkel W.M., Lillard T.S., Karaoli T., Gregory S.G., Mohammadi N., Epstein L. (2019). Early nurture epigenetically tunes the oxytocin receptor. Psychoneuroendocrinology.

[33] Unternaehrer E., Meyer A.H., Burkhardt S.C., Dempster E., Staehli S., Theill N., Lieb R., Meinlschmidt G. (2015). Childhood maternal care is associated with DNA methylation of the genes for brain-derived neurotrophic factor (BDNF) and oxytocin receptor (OXTR) in peripheral blood cells in adult men and women. Stress.

[34] Simons R.L., Lei M.K., Beach S.R., Cutrona C.E., Philibert R.A. (2017). Methylation of the oxytocin receptor gene mediates the effect of adversity on negative schemas and depression. Dev. Psychopathol..

[35] Lancaster K., Goldbeck L., Puglia M.H., Morris J.P., Connelly J.J. (2018). DNA methylation of OXTR is associated with parasympathetic nervous system activity and amygdala morphology. Soc. Cogn. Affect. Neurosci..

[36] Puglia M.H., Lillard T.S., Morris J.P., Connelly J.J. (2015). Epigenetic modification of the oxytocin receptor gene influences the perception of anger and fear in the human brain. Proc. Natl. Acad. Sci. USA.

[37] Wang F., Pan F., Tang Y., Huang J.H. (2021). Editorial: Early Life Stress-Induced Epigenetic Changes Involved in Mental Disorders. Front. Genet..

[38] Puglia M.H., Connelly J.J., Morris J.P. (2018). Epigenetic regulation of the oxytocin receptor is associated with neural response during selective social attention. Transl. Psychiatry.

[39] Porcher C., Medina I., Gaiarsa J.-L. (2018). Mechanism of BDNF Modulation in GABAergic Synaptic Transmission in Healthy and Disease Brains. Front. Cell. Neurosci..

[40] Kasarpalkar N.J., Kothari S.T., Dave U.P. (2014). Brain-Derived Neurotrophic Factor in children with Autism Spectrum Disorder. Ann. Neurosci..

[41] Ma K., Taylor C., Williamson M., Newton S.S., Qin L. (2023). Diminished activity-dependent BDNF signaling differentially causes autism-like behavioral deficits in male and female mice. Front. Psychiatry.

[42] Roth T.L., Sweatt J.D. (2011). Epigenetic marking of the BDNF gene by early-life adverse experiences. Horm. Behav..

[43] Daskalakis N.P., De Kloet E.R., Yehuda R., Malaspina D., Kranz T.M. (2015). Early Life Stress Effects on Glucocorticoid—BDNF Interplay in the Hippocampus. Front. Mol. Neurosci..

[44] Forsberg S.L., Ilieva M., Maria Michel T. (2018). Epigenetics and cerebral organoids: Promising directions in autism spectrum disorders. Transl. Psychiatry.

[45] Jones R. (2013). Trauma and stress, from child to adult. Nat. Rev. Neurosci..

[46] Hill K.T., Warren M., Roth T.L. (2014). The influence of infant-caregiver experiences on amygdala Bdnf, OXTr, and NPY expression in developing and adult male and female rats. Behav. Brain Res..

[47] Kinlein S.A., Wilson C.D., Karatsoreos I.N. (2015). Dysregulated Hypothalamic–Pituitary–Adrenal Axis Function Contributes to Altered Endocrine and Neurobehavioral Responses to Acute Stress. Front. Psychiatry.

[48] Liston C., Gan W.-B. (2011). Glucocorticoids are critical regulators of dendritic spine development and plasticity in vivo. Proc. Natl. Acad. Sci. USA.

[49] Makris G., Agorastos A., Chrousos G.P., Pervanidou P. (2021). Stress System Activation in Children and Adolescents with Autism Spectrum Disorder. Front. Neurosci..

[50] Gutierrez-Arcelus M., Ongen H., Lappalainen T., Montgomery S.B., Buil A., Yurovsky A., Bryois J., Padioleau I., Romano L., Planchon A. (2015). Tissue-Specific Effects of Genetic and Epigenetic Variation on Gene Regulation and Splicing. PLoS Genet..

[51] Schaafsma S.M., Gagnidze K., Reyes A., Norstedt N., Månsson K., Francis K., Pfaff D.W. (2017). Sex-specific gene–environment interactions underlying ASD-like behaviors. Proc. Natl. Acad. Sci. USA.

